# Bovine Tuberculosis in Britain and Ireland – A Perfect Storm? the Confluence of Potential Ecological and Epidemiological Impediments to Controlling a Chronic Infectious Disease

**DOI:** 10.3389/fvets.2018.00109

**Published:** 2018-06-05

**Authors:** A. R. Allen, R. A. Skuce, A. W. Byrne

**Affiliations:** Veterinary Science Division, Agri-Food and Biosciences Institute, Belfast, United Kingdom

**Keywords:** *Mycobacterium bovis*, Britain and Ireland, eradication, persistence, epidemiology

## Abstract

Successful eradication schemes for bovine tuberculosis (bTB) have been implemented in a number of European and other countries over the last 50 years. However, the islands of Britain and Ireland remain a significant aberration to this trend, with the recent exception of Scotland. Why have eradication schemes failed within these countries, while apparently similar programs have been successful elsewhere? While significant socio-economic and political factors have been discussed elsewhere as key determinants of disease eradication, here we review some of the potential ecological and epidemiological constraints that are present in these islands relative to other parts of Europe. We argue that the convergence of these potential factors may interact additively to diminish the potential of the present control programs to achieve eradication. Issues identified include heterogeneity of diagnostic testing approaches, the presence of an abundant wildlife reservoir of infection and the challenge of sustainably managing this risk effectively; the nature, size, density and network structure of cattle farming; potential effects of *Mycobacterium bovis* strain heterogeneity on disease transmission dynamics; possible impacts of concurrent endemic infections on the disclosure of truly infected animals; climatological differences and change coupled with environmental contamination. We further argue that control and eradication of this complex disease may benefit from an ecosystem level approach to management. We hope that this perspective can stimulate a new conversation about the many factors potentially impacting bTB eradication schemes in Britain and Ireland and possibly stimulate new research in the areas identified.

## Introduction

Bovine tuberculosis (bTB) caused by *Mycobacterium bovis* is a zoonotic disease, primarily affecting livestock, which is of economic importance to the European Union (EU) due to its impact on trade. Indeed, at the inception of the European project, as the European Economic Community (EEC), the first legal initiatives were taken to combat the disease in 1964 with the drafting of council directive 64/432/EEC (1). The latter document foresaw that there was a requirement for animal health legislation to underpin intra-community trade in livestock and introduced the concept / definition of being “officially tuberculosis free” (OTF), defined as the percentage of herds confirmed as bTB infected not exceeding 0.1% per year for six consecutive years (2). The legislation also defined the goal of the EEC to be disease eradication as opposed to control. Further legislation followed which enshrined the need for member states of the EEC to fund and facilitate test and slaughter schemes for the purposes of bTB eradication (3). In many member states, eradication programmes proceeded effectively, resulting in the granting of OTF status to Denmark in 1980, the Netherlands in 1995, Germany and Luxembourg in 1997, Austria in 1999, France in 2001 and Belgium in 2002 (2). Other states were granted OTF status upon joining the EU – Finland and Sweden 1995 and Czech Republic 2004 (2). In 2009, Poland and Slovenia also attained OTF status, whilst non-member state Norway was recognised as meeting all EU standards around OTF status (4).

Against this backdrop of successful eradication is the contrasting situation observed in the islands at the western fringe of the European continent – Britain and Ireland. Despite dramatic initial success in controlling bTB, England, Wales and Northern Ireland have suffered increasing incidence since the late 1980s. The Republic of Ireland experienced a relatively less dramatic initial reduction in incidence and continues to exhibit an ongoing problem in eradicating bTB, however, recent figures suggest that the situation has stabilised. Scotland is the notable exception, having been granted OTF status in 2009 (4). Data from 2009 indicated that 5–6% of herds from both islands tested positive for the presence of *M. bovis* (4). A contributing factor to the rise in herd incidence in England and Wales can be attributed to the suspension of bTB testing during the 2001 outbreak of foot and mouth disease (5, 6). However it has been recognised that even before the foot and mouth epidemic, bTB herd incidence was on the rise and foot and mouth disease merely exacerbated an already existing problem consistent with an upward trend in incidence since 1986 (6). Indeed, data from the Department of the Environment Food and Rural Affairs (DEFRA) in Britain indicates that ‘*since 2003 the total number of new bTB breakdowns identified (every quarter) in GB has been doubling at a rate of every 10 years. Prior to the Foot and Mouth Disease epidemic of 2001, the doubling rate was every 5.2 years’* (7). More recently, England, Wales and Northern Ireland have exhibited a rise in herd incidence whilst the Republic of Ireland has experienced a fall (6). Whilst mainland Europe still has a substantial bTB problem in the Iberian peninsula (Spain herd incidence 1.4% in 2009), this pales in comparison to the problems observed in the UK and Republic of Ireland (4).

This begs the question – why, in comparison to continental neighbours, have the territories of the United Kingdom and Republic of Ireland struggled to eradicate bTB? In Europe, diagnostic testing using some variant of the tuberculin skin test methodologies is highly standardised (8–10) following international protocols laid down by EU Council Directive 64/432. Debate around the efficacy of the skin tests are an ongoing matter of concern, with traditional epidemiological approaches estimating wide ranges of sensitivity of 52–100% - median 80% (8). More recently, Bayesian non-gold standard methods applied to data from Ireland have suggested skin test sensitivity is in the region of 50–60% (11), which has been further supported by a Bayesian meta-analysis from studies in the literature from 1934 to 2009 (12). Such relatively poor diagnostic performance may explain a failure to eradicate disease since many truly infected animals will be mis-classified as disease free. However, similar testing schemes using standardised reagents, presumably with similar test performance characteristics, have been used across Western Europe and indeed other parts of the world such as Australasia (13, 14), resulting in disease freedom. Furthermore, the performance improves at the herd-level as a screening test to identify infection, depending on the herd-size (numbers tested) and true within-herd prevalence (15). Also, once infection is identified, supplemental testing with more sensitive tests can be used to clear the within-herd infection (16). One could come to the perhaps overly simplistic conclusion therefore, that even with a poor individual test sensitivity, eventual eradication can be attained. However if this is true, what is confounding progress in Britain and Ireland? Undoubtedly, despite widespread standardisation in the basic diagnostic approach of using injectable tuberculins, there are individual differences in the application of eradication programmes subject to the variations of differing national policies, politics, behaviours and country specific factors (6). Indeed some of these socio-political factors may be some of the most important factors to account for the difficulties encountered in Britain and Ireland (17). Even with such heterogeneity of approach across time and national boundaries, it remains startling that particularly in Britain, which came close to achieving eradication in the 1960 and 1970s **(**8, 18), bovine TB is resurgent (as discussed above). Therefore, alongside issues of differing diagnostic test application protocols, we propose it is also timely to consider other potential additional factors whose current impact is unknown, but which may be additively preventing progress towards eradication.

Specifically, we hypothesise that there may exist a convergence of detrimental risk factors unique to GB and Ireland that is undermining the bTB eradication effort in these territories. If certain factors do contribute to this hypothesised “perfect storm” underpinning a failure to eradicate bTB, what are they likely to be? Below we discuss some of the likely ecological and epidemiological candidates given current knowledge. These proposed factors are not meant to be a definitive or exhaustive list; indeed we fully recognise that there may be many “unknown unknowns”. Rather our intention is to attempt to address the current impasse in bTB control in these islands by adopting a novel perspective which seeks to address the likely multi-factorial problems which afflict our national eradication programmes. We hope that in so doing, we can start a debate on how this *perfect storm* can be investigated and addressed through innovative approaches and methodologies.

## The factors

### Heterogeneity in bTB Diagnostic Approaches and Control Programmes

In the late nineteenth century, tuberculins, derived from the culture filtrate of TB causing bacilli (10), were initially produced as potential therapeutic agents by the discoverer of the tubercle bacllius, Robert Koch (10). Their lack of efficacy for this particular task, was superseded by the discovery they could be used in the diagnosis of tuberculosis (8, 10). In the twentieth century it was discovered that intradermal injection and measurement before and after of skin thickness could be used to detect the delayed type hypersensitivity (DTH) reaction, indicative of bTB infection (8, 10). Since then, “repetitive use of tuberculin tests remains the basis of all bTB control programs to this day” in areas with endemic disease (10), following strict standards laid down by EU council directive 64/432.

Despite such standardisation, there remain differences in application of skin tests and in the programs they support across the EU. We address an overview of these below:

#### Different Tuberculins and Divergent Potencies

Tuberculin potency for use in all skin tests used in Europe is tightly regulated by EU Council Directive 64/432. It is however recognised that changes in manufacturing and production procedures can result in batch to batch variation in potency (10). Indeed, even with strict regulation, tuberculins of lower potency have been released before (10). Such batch to batch variation and low potency would affect sensitivity of the skin test by reducing the DTH response / skin swelling that underpins diagnosis. Could such heterogeneity underpin the divergent outcomes in Britain and Ireland’s TB control programmes compared to mainland Europe? An understanding of the history of tuberculin production may help to partially address this issue. Prior to 1975, the UK produced tuberculin derived from three *M. tuberculosis* strains, which was used in both Britain and Ireland [(8), M. Good personal communication]. Use of this tuberculin, derived from the human pathogen, coincided with the lowest prevalence of bTB in GB suggesting that the testing scheme was very effective. In 1975, both the United Kingdom and Republic of Ireland switched from using an *M. tuberculosis* derived PPD to one derived from the GB AN5 strain of *M. bovis* (8) as this exhibited superior sensitivity and specificity to the *M. tuberculosis* PPD (8, 19, 20). During the period after the 1970s, England and Wales experienced the well documented rise in bTB prevalence despite using this apparently superior Weybridge tuberculin (6). In 1980 the Republic of Ireland switched to using tuberculin produced from the AN5 strain in Lelystad (M. Good personal communication) with the UK following suit in 2008 (21). Downs et al. (21) went on to compare Weybridge and Lelystad tuberculins for bTB breakdown data in GB between 2005 and 2009, finding that the Weybridge formulation exhibited a slightly higher sensitivity and lower specificity. Given this data, in the context of increasing prevalence of bTB in the UK, the Weybridge tuberculin could have resulted in the reduction of false negative animal / herd detection whilst increasing the false positive rate – a combination in conflict with the hypothesis that tuberculin differences underpin the prevalence rise in GB. Whilst it is impossible to know the quality of all Weybridge tuberculin batches produced between 1977 and 2009, it is pertinent to note that batch to batch variation is not just a feature of GB production from the 1970s onwards (21, 22).

#### Differing Test Formats Across Europe

The most obvious difference is related to the exact test used. In Continental Europe, the single intradermal test (SIT) / cervical intradermal test (CIT) is used, involving the inoculation of *M. bovis* derived purified protein derivative (PPD) to detect skin thickness increases indicative of infection (8, 9). Conversely, in Britain and Ireland, owing to problems with environmental members of the* Mycobacterium avium* complex of bacilli cross reacting with *M. bovis* PPD and reducing specificity (increasing false positives), a comparative test is used (8). The single intradermal comparative cervical test (SICCT) injects both *M. bovis* and *M. avium* derived PPD into separate locations of an animal’s neck, and uses the resulting difference in skin thickness between both sites as a diagnostic metric (8). The SIT has been noted to have an increased sensitivity (fewer false negatives) compared to the SICCT (23): SIT sensitivity range 80.2–91.2%; SICCT sensitivity range 52–93.5% (8). It is possible that this difference in sensitivity in the test used in Britain and Ireland may contribute to the comparative difficulty in clearing infection these islands experience, compared to continental neighbours. However, if this were the sole cause of the latter stark difference, it fails to explain why the Republic of Ireland and the United Kingdom exhibit such divergent contemporary and historic herd prevalences (6) despite having used similar tuberculin preparations (see above).

#### Differing Approaches to bTB Programs Across Europe

In continental European countries, which do not have the same problem as Britain and Ireland, bTB testing programs can have radically different outcomes. For example herd de-population upon the detection of reactor animals was common in France (9) – effectively using skin test as a herd screening test (8). In the continental context however, there is now a move away from whole herd de-population measures because of the costs involved and also animal welfare concerns (9). Economic consequences of whole herd depopulation in Britain and Ireland are of even greater significance owing to the much higher infection prevalence in these regions, and consequently are generally rarely deployed (with notable exceptions: (24, 25). A movement towards more pre-movement testing, to prevent spread of infection to new areas, has been proposed as an alternative to depopulation (9), Pre-movement testing in the Republic of Ireland was a required part of the TB eradication scheme up until 1996 (20). Since then, an evaluation of reviving the practice in 2005 revealed no significant cost benefit, with the suggestion being that very few outbreaks were being caused by onward movement of animals (20). In Britain, pre and post movement testing was introduced in 2006 (APHA, 2017) at direct cost to individual farmers. In Northern Ireland, pre-movement testing has been considered (26), but is currently not an active part of the eradication scheme.

Deployment of gamma interferon, as a higher sensitivity ancillary test, in Britain and Ireland is variable – in Northern Ireland, participation in testing has been voluntary, with no statutory powers in place to remove test positive animals (16). In the Republic of Ireland and in Britain gamma interferon is now conventionally used in problematic herds to increase sensitivity in an attempt to clear infection, but their application and the basis for animal removal has varied widely (6).

Other European countries such as Switzerland also use the practice of taking inconclusive reactors from skin test positive herds (9); however it should be noted, Switzerland who undertook biennial testing (1960–1980), now resort to passive abattoir surveillance from 1980 onwards (9). Such inconclusive reactor animals have been shown to be at elevated risk of developing infection in the future (27) and could potentially lead to the retention on farm of high risk animals. The economic cost in removing all inconclusive reactor animals is an order of magnitude greater in countries like Britain and Ireland which have a higher disease prevalence.

Compartmentalisation of regions within European countries, based on relative prevalence has been used to contain infection and prioritise resources (2). However, the restrictions used to control spread of infection across these has not been implemented uniformly. Over a number of years, Italy compartmentalized some regions with higher prevalence (28), applying greater movement restrictions between regions on the basis of risk. In Britain, compartmentalization has only been implemented in recent years, and has does not completely prevent movement – relying on pre and post movement tests (29, 30). It is notable, this regionalization approach is not effective in places which have widespread infection without a single locus, such as the island of Ireland (31).

 It is conceivable that the program differences discussed above have compounded the ongoing problem Britain and Ireland have with bTB, however it is currently difficult to quantify the magnitude of these impacts.

#### Summary

In summary, these differences in tuberculin potency, application of skin test formats and heterogeneity in downstream choices in program management could have had a divergent effect on the bTB outcomes observed in Britain and Ireland vs Continental Europe. However, it is extremely difficult to untangle their relative importance (as is the case for all of the hypothesised factors), especially against the background of such differing epidemiological (and ecological) contexts. Future research efforts, including “big data”, could assess differing interventions in settings with similar prevalence. Such approaches are predicated on better data harmonisation and sharing.

## Wildlife

### Wildlife Hosts Are a Significant Impediment to Eradication

The presence of wildlife hosts of bTB has been found to be a major impediment to eradication in a number of countries worldwide [e.g., Michigan, USA, New Zealand, UK and Ireland; (32)]. Spill-back infection from wildlife to cattle can seed infection into cattle herds (33). In Michigan the wildlife host is the white tailed deer [*Odocoileus virginianus*; (34)], while in New Zealand there is a multi-host problem with the most significant reservoir being the non-native brushtail possum [*Trichosurus vulpecula*; (14)]. In continental Europe, recent research has suggested that wild boar may act as a reservoir of infection, causing increased risk to cattle herds in parts of France and Spain (35–37). Deer may be a widespread, but relatively localised, problem in a number of countries across Europe (38). It has been suggested that European badgers may also play a role in the epidemiology of bTB in cattle in Spain and France (39, 40), however, it is only in Britain and Ireland where there is strong evidence of their impact on the control of bTB in cattle (41–44).

### Evidence That Badgers Are Implicated in the Epidemiology of bTB in Cattle - Culling

Badgers are a host species for *M. bovis*, and have been implicated in the epidemiology of cattle bTB in UK and Ireland (41, 42, 44)]. Culling trials have demonstrated significant reduction in risk to cattle herds in areas where badger densities were significantly reduced (41–44). The magnitude of this effect has been shown to be larger in the ROI than in (Randomised badger cull) trials in GB [compare (43, 45) with (41, 42)]. In GB, badger culling was associated with a temporarily increased risk also to herds found at the periphery of cull sites (41). This was hypothesised (the “perturbation effect” hypothesis) to be as a result of increasing frequency-dependent transmission amongst badger populations, causing increased spill back infection to cattle herds (41). This suggests that badgers can play a significant role in spilling back infection to cattle over short duration. However, this peripheral increased risk was transient [<2 years post-cull (46)]; and was not demonstrated during badger cull trials or government-led culling operations in ROI (43, 44, 47, 48). The apparent beneficial effects of proactive culling to farms in cull areas have been maintained for up to 5–11 years after GB cull trials (49–52), and up to 10 years post-cull trial in ROI (53).

### Cattle and Badgers Share bTB Pathogen Strains Which Cluster in Time and Space - Strain Typing and Whole Genome Sequencing

Strain typing of *M. bovis* has demonstrated that both badgers and cattle share similar strains with geographic clustering across hosts indicative of interspecific transmission at local scales (48, 54–56). The best evidence for this ongoing transmission has been demonstrated at the genomic level (57, 58). Biek et al. (57) demonstrated that at the farm level, badgers and cattle shared the same or highly similar pathogen sequence type consistent with frequent and recent transmission events – however, the direction of transmission could not be established during that study. It is likely that transmission occurs in both directions (53, 59, 60); however, the force of infection may be greater from badgers-to-cattle than cattle-to-badgers owing to the continual removal of infected cattle through test-and-slaughter (61–63). It is conceivable there may be regional variation in the latter as a result of animal and wildlife densities. Furthermore, culling experiments (see above) have demonstrated that the cycling of infection can be interrupted with beneficial effects for reducing bTB prevalence in the target host population.

### Britain and Ireland Have Higher Average Badger Densities Than Elsewhere in Europe

The islands of Britain and Ireland have the highest average recorded density of badgers compared to any other country in Europe (64). Median badger densities across badger study sites suggest a median density of 4.3–5.4 badger km^−2^ for the British Isles, while studies from across Europe suggest that median badger densities are 0.29–0.55 badger km^−2^ [(64, 65), Byrne, unpublished]. Furthermore, in England and Wales there has been a significant increase in badger social group and population densities in recent decades (66, 67). These figures mask the wide variation in density at lower spatial scales (66, 68–70) – for example, badger densities in Spain can vary from <0.3 to 3.4 badgers km^−2^ across habitat types (71). Similarly, badger densities in Ireland can vary from 0.7 badgers km^−2^ in poor upland habitat (72); in ideal conditions on a wooded island, densities up to 37 badgers km^−2^ have been recorded (64). However, what is important is that badgers benefit from a benign temperate climate in the British Isles (73, 74), and have thrived in areas where woodland and pasture abound (66, 68–70). These habitats can maintain badgers at mean densities of 3–5 badgers km^−2^ in Britain and Ireland (64, 75) and are also the contact point for potential direct and indirect interaction between badgers and grazing cattle (76). While badger density *per se* may not be related directly to risk in a linear fashion, large-scale epidemiological studies in the Republic of Ireland (53), Northern Ireland (77) and in Great Britain (78) have found significant and positive associations between metrics of badger density and increased bTB herd breakdown risk.

There is considerable variation in the societal attitudes to the management of wildlife across Europe [e.g., see (65, 79–84)], and this results in significant variation in the actual management practices implemented across Europe. This may relate to the apparent conflict between conservation, animal welfare, and management goals, as well as cultural differences in the acceptability of pursuits such as hunting. Hunting is more common, and arguably more socially acceptable in many continental European countries [e.g., (82, 83)] than it is in the UK and Ireland. Badger hunting is widespread and relatively intense in a number of countries across continental Europe (65), despite the badger being listed under the Bern convention. In Germany, the annual recorded hunting bag for badgers has been between 50–70,000 per annum, within an increasing trend in the hunt bag in recent years – for example, the bag for 2016 was 71,168, a 11.98% increase from 2015 (85). Similarly, in Finland 8,600–14,000 badgers have been reported in the national hunting records per annum (86, 87) and an increasing trend is reported in Poland where recent game bags are in excess of 4,000 badgers (88). Badger hunting is common and widespread in France, though there are limited available data on the national badger status (89), but hunters have been used recently in bTB outbreaks to sample badgers (39, 90). In Britain and Ireland, the badger has been protected by legislation since the 1970’s. It is likely that this protection status has had beneficial effects on population size (68, 70, 91, 92) and may have influenced the considerable variation in the estimated densities of badgers across European countries, and between the British Isles and continental Europe. Furthermore, this broader issue of the “palatability” of wildlife management within society, and the relationship between this effect and the interventions undertaken may have been a significant factor in the bTB epi-system within the UK and Ireland (93).

### Badgers Exhibit Significant TB Prevalence

Prevalence of *M. bovis* infection in badger populations may be sought as base-line data, although it is likely to vary by region and over time and is recognised as being difficult to quantify accurately (94–96). Standard pathology investigations have limited sensitivity (42, 97, 98) with the result that prevalence is likely to be underestimated.

Recent investigations indicate that more detailed post-mortem examinations result in the detection of microscopic lesions that would otherwise evade detection by standard procedures (97–99). Badgers killed in road traffic accidents (RTA) have proven to be a useful source of data in attempting to determine badger TB prevalence at a county-wide scale (42, 56, 100). The ISG reported that standard post mortem examination revealed that 15% of GB RTA badgers had TB (42). The ISG cautioned, however, that at a localised level below county size, owing to reduced availability of RTA badgers, this method may not be sufficient for surveillance (42). Similar RTA data collected in NI indicated that ~15% of badgers were infected (101). In GB, the ISG reported that in proactive cull regions, 16.6% of badgers were tuberculous (42) whilst in reactive cull regions this figure was 14.9% (102).

Similarly, studies in the ROI indicated that, by the standard protocol, culled badger TB prevalence was 12.1% (98) and largely in agreement with RTA figures. More thorough post mortem examination of culled badgers led to the detection of an increased number of infected animals. Cranshaw et al. (97) demonstrated that, in GB, proactively culled RBCT badgers had a true prevalence of TB infection of 24.2%. Similarly, in the ROI, more detailed post-mortem examination of culled badgers from across the country revealed a prevalence of 36.3% (98). However, other studies found higher prevalence up to 43%, indicating the variation in estimates depending on sampling and laboratory methods (103). Using cage trapping, anaesthesia and live sampling of badgers Drewe et al. (104) used latent class analysis to estimate the outcome of multiple tests on live badgers (culture, gamma interferon and Stat-Pak ELISA), in the absence of a perfect gold standard diagnosis. Sensitivity of diagnostic testing was estimated at ~93% and badger TB prevalence was estimated subsequently as 20.8% in Woodchester Park, Gloucester (104). Intra-regional, inter-regional and temporal differences in badger TB prevalence are to be expected owing to the potential differences in ecology and population dynamics of both cattle and badgers in different areas as illustrated recently in the ROI (105). Indeed, Byrne et al. (74) demonstrated large spatial variation in badger infection risk based on a sample of over 5,000 badgers across the Republic of Ireland. Using standard PM techniques and bacteriological culture confirmation, there was an order of magnitude difference in the worst infected counties to the lowest prevalence counties. Furthermore, there was a significant decrease in prevalence from 26 to 11% positive, at a period where TB was declining in the local cattle populations.

Regardless of “true” prevalence, these studies indicate that a significant component of the badger population across the UK and Republic of Ireland is infected with *M. bovis*, where bTB is also prevalent in cattle.

## Cattle and Husbandry

### Britain and Ireland Have Some of the Highest National Cattle Densities in Europe

As has been described above for the European badger, the density of host organism available for infection by the TB causing bacilli seems to be of critical importance to ongoing transmission of disease and persistence. By analogy to bTB, Human tuberculosis, caused by *M. bovis*’ close relative, *Mycobacterium tuberculosis,* is typically associated with overcrowding in confined spaces (106, 107). It is not surprising therefore that as with badgers, cattle densities will probably have an influence on bTB transmission dynamics. The countries that make up the islands of Britain and Ireland have notably high cattle densities in comparison with other European countries. In 2010, out of 27 countries within the EU, Northern Ireland had the highest mean cattle density of any country at 112 cattle per km^2^ (108, 109). The Republic of Ireland was third (84 cattle per km2), with Wales and England ranking 6 and 7th respectively (54 and 40 cattle per km2). Scotland, who are now officially bTB free, are ranked 13th with a mean density of 22 cattle per km2. To illustrate the differences amongst countries, the national herds are large relative to their area in Britain and Ireland, for example, both the Republic of Ireland and Spain have approximately 6 million cattle (109), yet Spain is 7.2 times the size of ROI (504,645 Spain/70,273 ROI km^2^). Research suggests that both the size of herds [e.g., (53)] and the intensity of farming (110) can be associated with increased risk of bTB breakdown in endemic countries [for reviews see (54, 111, 112)]. Larger herds may constitute greater risk as it may be more difficult to clear infection, once identified within the herd, due to the poor sensitivity of skin tests (113). The density of cattle within farms can be a proxy measure for the intensity of agricultural production, and has been associated with increased risk of bTB (110). At a macro-scale the risk of bTB increases with increasing intensity (111), primarily due to closer proximity between animals and potential infectious contacts (110, 112). In Britain and Ireland there has been a move towards intensification, with a trend towards larger farm sizes, yet a decline in the absolute number of farms (113, 114). Recent changes in dairy production at the EU level may exacerbate this pattern in the future. However, interestingly, Acevedo et al. (115) did not find a relationship between host density and bTB prevalence when investigating European island as discrete bTB ecosystems.

### Britain and Ireland Have Farming Characteristics That May Cause Difficulties in Managing Infectious Disease

Trade is a significant characteristic of cattle farming in the UK and Ireland, with significant patterns of movement that transcend national boundaries (116, 117). Indeed, for example, in 2015 there were 55,285 live animal exports from ROI to Northern Ireland (118). Gilbert et al. (116) showed that, at a GB level, there were significant flows of animals traded over long distances, and also showed that movement metrics were a significant risk factor for bovine TB. Ashe et al. (117) visualised the movement of a cohort of animals from one county in one year in Ireland; a remarkable pattern of movement that encompassed all regions of the island was apparent. However, cattle movement and trade is a scale dependent phenomenon. While long distance movements occur and can potentially link disparate areas epidemiologically, the majority of trade moves are local (119) – the movement kernel is long tailed (120). Recent analysis of trade networks in Northern Ireland has demonstrated that farms are extremely well connected, forming a robust network that is resistant to random and targeted node removal. Essentially, this indicates that the interconnectedness of this herd network makes it difficult to manage spread on an individual basis.

Small movement networks can contribute to the local risk of bTB (121), however they also may explain the strong clustering of pathogen genotype patterns at a local level in Northern Ireland (54). Furthermore, there is a phenomenon of farm fragmentation, whereby herds are made up of a number of spatial fragments. These fragments can have large footprints (122), relative to the home premises, allowing for increased exposure to neighbours or environmental reservoir risk. The long-established practice of seasonal rented grazing, known in Northern Ireland as “conacre,” adds to the potential impacts of fragmentation. Furthermore, there are little data available to assess within herd movements of animals – a potential for dispersal of infection both to neighbouring herds, but also spread of infection into the environment, including wildlife hosts. The movement of animals, the spreading of slurry and the sharing of farm equipment could all increase the likelihood of maintenance of TB (92), furthermore the constituent nodes within these networks (e.g., specific farms, marts, auctions) can have disproportionate effects on diseases spread (120, 123). On the other hand, the fact that islands are disconnected to the continent, raises the perspective that this insularity could prove beneficial towards the longer term control of the pathogen (115). Currently, there is a lack of harmonisation of data pertaining to animal movements within Western Europe, to allow direct comparisons between EU member states in terms of network structure and connectivity. Attaining this harmonisation should be a major research goal going forward. Anecdotally, the very dense within and between herd movement networks in Ireland and the UK, are different compared to the rest of Europe. However, without detailed comparative data, this makes direct comparisons challenging.

## The Pathogen: *M. Bovis*

### *M. Bovis* Strain Heterogeneity

The population genetics of the *M. bovis* bacillus in these islands is relevant to investigate the current epidemic and is an ongoing source of interest for many researchers. From a phylo-geographic point of view, such research can inform on the population history and can potentially inform on probable routes of entry into Britain and Ireland in the distant past (124). From a more practical and less academic point of view, phylo-geographic differences in pathogen demographics and evolution may have an outcome that is of importance to disease control and epidemiology. Such region-specific evolution and adaptation can result in differing pathogen phenotypes that result in differing disease outcomes and dynamics as has been well-documented for *M. tuberculosis* infection in humans (125–128). For example, in Vietnamese populations, specific strains of a Beijing lineage of *M. tuberculosis* have been observed in a number of cases to result in a meningeal form of the disease, rather than the expected pulmonary disease pattern (129). Indeed the Beijing lineage of *M. tuberculosis* is recognised as exhibiting a hypervirulent phenotype distinct from other phylo-geographically distinct lineages (130). The latter evidence indicates that certain lineages of a tuberculosis-causing bacillus can vary in their phenotype in epidemiologically meaningful ways. So, the obvious question arises, could something similar happen with bovine tuberculosis caused by *M. bovis?*

Consequently, it is pertinent to attempt to understand the population structure and history of *M. bovis* in Britain and Ireland. The extant *M. bovis* population is almost exclusively dominated by a single clonal complex – the Eu1 clonal complex (124). This is indicative of a genetic bottleneck occurring at some point in the pathogen’s history on these islands, an event which led to a contraction in diversity, resulting in greater homogeneity. Such bottlenecks are features of clonal pathogens like *M. bovis* (131). Whether this bottleneck occurred at the time of colonisation or subsequently is unknown. While this Eu1 lineage is present on other parts of Western Europe it is nowhere near as dominant as it is on these islands (124). Eu1 is also found globally among former trading partners and members of the British Empire, suggesting wider dissemination during colonial times (124). It has been shown that this Eu1 lineage spread throughout Britain and Ireland, leading to a homogenised *M bovis* population potentially brought about by the free movement of infected animals between territories (132). More recent cattle movement controls as part of national TB eradication schemes may have subsequently isolated regions one from another and driven more local evolution of specific strain types (132). Previously, Smith et al. (133) had sought evidence to support a hypothesis that a test and slaughter bottleneck of the *M. bovis* population in the 1950 and 1960s may have constituted selection pressure for the evolution and clonal expansion of a “fitter” clone that exhibited some form of advantage with respect to evading the test and slaughter scheme. Quite what the latter advantage could be, if it existed, is still a matter of debate. An ability to evade diagnostic testing is one possibility whilst invasion of / adaptation to a new host / niche such as a wildlife reservoir is another possibility (133).

The host range of the TB- causing bacilli is an intriguing puzzle (134). The human pathogen, *M.* tuberculosis, disseminates in human populations but appears not to be transmissible between non-human animals (134–136). Conversely, *M. bovis* appears to be able to disseminate among many non-human animal species, but humans are generally a dead end host (134–136). However, there is an interesting exception. Recently, Gonzalo Ascenzio *et al.* (137) demonstrated that a subtle mutation in the virulence genes of *M. bovis* can cause it to freely disseminate in humans. This is evidence that a small genetic change in a pathogen can radically expand maintenance host range. Could the Eu1 lineage of *M. bovis* have undergone a similar transition to become a better host generalist? It is noticeable that wherever Eu1 strains are found around the globe, there is a wildlife reservoir problem (124, 134). However, some caution is required here. This may just be an effect of recent demography and trade (124). The Eu1 lineage may just be a “lucky clone”, dispersed by chance events. Additionally, the countries which inherited its diaspora are mostly developed world nations likely to have good disease surveillance infrastructure. Therefore, perhaps apparent increased propensity for wildlife adaptation is purely confirmation bias? The fact that many of these countries have had much greater success in bovine TB eradication than Britain and Ireland is also perhaps indicative that there is nothing obviously fitter about the Eu1 clonal complex, and that the wide dissemination of this lineage may purely be a matter of demography and international trade (124). Additionally, other European lineages of *M. bovis*, distinct from Eu1, have been observed to infect cattle and wildlife populations in Spain (138), Portugal (139) and France (140). However, in the absence of empirical comparisons between multiple *M. bovis* lineages, the pathology they induce across multiple hosts and their epidemiological characteristics, it is perhaps premature to rule out the hypothesis of the Eu1 lineage being in some way fitter. This bears further investigation (134). It is not inconceivable that whilst multiple *M. bovis* lineages have similar host ranges, the relative efficacy and virulence within similar hosts may be different owing to genotypic and phenotypic divergence as has been seen with *M. tuberculosis* lineages (127). Similarly, whilst the global diaspora of Eu1 *M. bovis* strains arising from historical trade and colonialism (124) are undoubtedly genetically similar, there remains the potential for region specific evolution since introduction. Different ecological contexts and applications of control schemes could have resulted in phenotypic divergence from a similar ancestral stock of bacilli.

Further work looking for an *M. bovis* strain phenotype in Northern Ireland has yielded limited evidence of an advantageous adaptation with regard to ability to evade detection. Wright et al. (141) demonstrated that field isolates of differing strain type exhibited no significant difference in response to the tuberculin skin test at the animal level. Allen et al. (132) raise the caveat that Wright *et al*’s study was confined solely to Northern Ireland which contains strains from only the Eu1 lineage, and a geographically distinct sub population of Eu1 at that. Given the likely genetic homogeneity, would one reasonably expect to find stark differences in disease outcome / pathogen phenotype in such a setting (132)? Ideally, comparison of the epidemiological characteristics of strains extant in the recent past, predating test and slaughter schemes, within Britain and Ireland would also have been very interesting. However these strains are unavailable as their presence predates molecular characterisation and sample storage. Indeed, our knowledge of the *M. bovis* population in these islands is currently limited to that which is extant, and we have no definitive way of knowing whether Eu1 strains have always predominated or supplanted another lineage(s) of the bacillus. The fact that Eu1 strains were exported during the time of Empire suggests this lineage may have been at the very least, common for a considerable period of time in Britain and Ireland. Therefore any speculation on a fitter phenotype evolving within Britain and Ireland may be moot. Allen et al. (132) suggest that casting the net wider and comparing Eu1 to non Eu1 lineages in Western Europe or further afield may yield more fruit in this endeavour. In line with this hypothesis, it is perhaps telling that differences in disease outcome in *M. tuberculosis* have been observed at the level of major lineages – see previous. It is of note however that in a study, again confined to Northern Ireland and Eu1 only strains, Wright et al. (142) did find evidence for a difference in strain virulence, and Milne et al. (143) have observed that certain strains are associated with chronic, ongoing infections in certain herds over many years.

Given the contrasting views and evidence discussed above, the null hypothesis, in which there is nothing inherently “special” about *M. bovis* strains in Britain and Ireland, remains inherently plausible, but worthy of greater study. Much of the argument that Eu1 is not special seems to hinge on anecdote in the absence of data. Where empirical evidence is available, there are limitations in our ability to infer wider trends from geographically restricted findings as discussed previously. It would therefore in our view, be pertinent to attempt to address issues of pathogen lineage and its potential effects on bovine TB diagnosis and host range with well-designed studies and analyses. Regarding effects on TB diagnosis, we have already suggested above that intra lineage comparison across a wider geographic area is a better way to definitively settle this question. Overall then, it is our opinion that it would be better to empirically affirm or reject these hypotheses around evasion of diagnosis and host adaptation rather than dismiss them on the basis of gut feeling in the absence of hard evidence. If either or both hypotheses proved to be have some grounding in fact, then this could have implications for the application of control schemes in Britain and Ireland.

## Co-Infection – Effects on bTB Diagnosis

Co-infection dynamics are increasingly being recognised as a driver of the heterogeneous response of hosts to infection, and for persistence of diseases over time (144, 145). *Mycobacterium bovis* infection may be modulated by the presence of other infections (146–148), especially where severe infections immunocompromise the host. There is some evidence that *Mycobacterium bovis* infection progression can be impacted by viral infections such as Bovine Viral Diarrhoea (BVD), with the immunological response compromising tests used to disclose infected animals (149, 150), but see (151). Similarly, exposure to other infectious *Mycobacterium* species such as *M. avium paratuberculosis* (MAP or Johne’s disease) can confound the immunological diagnosis of bTB through cross-reactivity (151–153). Furthermore, environmental mycobacteria can also affect the performance of bTB immunological tests, for example *M. hiberniae* (154, 155). Some of these environmental mycobacteria have been closely associated with bogs and peaty soils and subsoils (156), which is a significant habitat type within the British Isles (157), and could potentially impact on tuberculin skin test performance. Indeed, the potential for cross reaction is one of the reason why in Britain and Ireland the comparative skin test is used (bovine and avian tuberculin), which is not the case in other jurisdictions where such cross-reactions are rare (8).

MAP is now endemic in Ireland and Britain (158, 159), and suffers from similar diagnostic problems to bTB. Recent research from Ireland has highlighted the potential nexus between MAP and bTB (151, 160, 161). MAP exposure can affect the correct diagnosis of bTB, hindering the disclosure of truly-infected animals. However, it should be noted that MAP is now widely distributed in Western Europe, and similar problems have been described there [e.g., Spain; (162)]. Britain and Ireland may be particularly vulnerable to interference owing to the fact both territories use the comparative tuberculin test.

Recently, liver fluke (*Fasciola hepatica*) infection has been associated with a negative impact on the disclosure of bTB using the experimentally-infected cattle model and SCIT testing (146–148, 163). The prevalence of fluke infection in GB has also been associated negatively with the probability of dairy herds breaking down for bTB after whole herd tests. The size effect was large, with an estimated under-ascertainment of 33% (148). Co-infection, therefore, represents a mask potentially hiding the true infection status of both animals and herds, making clearance and ultimately eradication very difficult.

Liver fluke is endemic in Britain and Ireland, with high prevalence of infection (148, 164–166). At farm level, prevalence has been estimated as 86% in Wales, 83% in Ireland and 48% in England (164, 165). At the animal level, >60% animals exhibit some evidence of fluke damage in the livers of slaughtered cattle in Ireland (167). Using surveillance data, Byrne et al. (166) showed that >60% of herds had some infected animals in Northern Ireland, while herd prevalence approached 100% where at least 100 animals were sampled over a three year period. Given the results of Claridge et al. (148), these levels of infection may have a significant impact on the disclosure of bTB-exposed animals using immunological tests like SCITT. However, recent research from Northern Ireland failed to show a large size effect of co-infection on tuberculin reactions from field data (161), but did find associations between fluke co-infection and TB pathology mirroring other studies (146, 161, 168). But how different are the British Isles than other countries in Europe in terms of fluke exposure?

Recent spatial analyses and comparative studies across Europe have suggested that there are significantly lower levels of infection in continental Europe than in the British Isles (169–171), with particularly high levels of infection in cattle throughout the island of Ireland (170).

The distribution and abundance of liver fluke in the environment is strongly affected by climate and habitat types, through exposure and survival of intermediate hosts (164). This is in part due to the wet, temperate climate within the north-western Europe (169, 170). The exposure of livestock in the British Isles is also affected by farming practice (field based grazing), soil type, high soil moisture level and the abundant access to fresh water sources (172, 173).

While recent research has found equivocal evidence for the mechanism (168), there has been no comparative analysis of data derived from low and high fluke prevalence areas (i.e., international comparisons). One suggested hypothesis in Ireland is that such a high proportion of animals are exposed that there is a general depression of tuberculin reaction sizes (161).

## Climate and Environment

### Climate Adaptation and Change - Effects on Fluke

Future forecasts of fluke infection risk paint a depressing picture for parts of Europe, with especially significant predicted increases in risk for Britain and Ireland (169, 174). These forecasts have been primarily derived from climate projections, which for the most part are suggesting that Britain and Ireland will become warmer and wetter on average, but also more climatologically variable. Fox et al. (174) forecast significant increases in fluke in all regions of the United Kingdom, with a projected epidemic for parts of Wales by 2050. Similarly, Caminade et al. (169) also forecast significant future risk for increasing fluke in Ireland and Britain up to the year 2080, but also increasing risk for north-western parts of continental Europe. This increasing parasitic risk, coinciding with the troubling emergence of fluckicide resistance, indicates that screening tests such as the SCITT and surveillance data based on post-mortem pathology for bTB, may become even less robust for disclosure of infected animals.

### Environmental Contamination with *M.bovis*

Despite recent advances in epidemiological analyses, molecular typing and whole genome sequencing of *M. bovis* (54, 57, 60), surprisingly little is known about the exact transmission mechanisms that spread infection within and between cattle and wildlife populations. Previously, owing to the work of UK government appointed ISG, who administered the Randomised Badger Culling Trial (RBCT) in Britain, it was assumed that direct contact between animals was required to facilitate disease transmission by aerosolised *M. bovis* bacilli leaving the respiratory tract  (42).The cited evidence for the primacy of this suspected transmission route was the preponderance of tuberculous lesions observed in the upper respiratory tracts of both badgers and cattle that underwent post mortem examination (42, 175). However, more recently, studies which made use of proximity logger collars fitted to sympatric (42) cattle and badgers in Northern Ireland and England, failed to detect very close contact between species that would facilitate direct respiratory transmission (176–179). These findings do not preclude the hypothesised existence of super-spreaders in cattle and badgers. The ISG however were sceptical that many such super spreader badgers existed (42). The latter may however be controversial, with some evidence of badger super-shedding in a high density English badger population (96), and indeed the precedent of super shedding in other wildlife species (180). The latter opinion, in concert with the apparent lack of meaningful direct contact between species, has raised the possibility that a contaminated environment may potentially be playing some indirect role in disease transmission between species (42, 177, 179, 181, 182). This hypothesis has been raised before, with studies in the past focusing on the potential role badger urine and faeces may play in contaminating soil, pasture and feed (76). Renal lesions have been observed to be the second most common type of tuberculous pathology in infected badgers in some earlier localised studies (183, 184). More recently, 13–14% of culled badgers exhibited such lesions in GB (185), with a similar 15% lesion presentation rate seen in Northern Ireland (101). Badger urine has been observed on occasion to contain 250,000–300,000 bacilli per millilitre (186, 187). Badger faeces deposited at latrines close to territorial boundaries have also been observed to be potential sources of *M. bovis* in the environment (188). These bacilli are believed to enter the GI tract via ingestion of respiratory mucus (51). In one gram of badgers faeces, 75 colony forming units have been observed (187). It is conceivable that badgers and cattle inspecting urine trails or faecal latrines left for territorial marking (75, 188) could aerosolise bacilli from these sources and seed a respiratory tract infection. Other prominent veterinary pathogens have been observed to be aerosolizable from an environmental source - *Coxiella burnetii*, which causes of Q Fever, has been observed to infect animals and humans exposed to contaminated wool (189) and *Mycobacterium avium paratuberculosis*, the causative agent in Johne’s Disease has been observed to be aerosolised in dust particles derived from bovine faecal material in animal housing (190). An intriguing recent study demonstrated that *M. canettii*, a pathogen predicted to be a common ancestor for the *M. tuberculosis* Complex, could produce pulmonary infection, indistinguishable from aerosol-mediated pulmonary infection, in mice fed spiked material (191).

A crucial question for the viability of this hypothesis is how long can *M. bovis* persist in the environment? Previously, bacilli in badger urine were observed to survive on pasture for ~3 days in the summer and ~14 days in the winter (186) potentially due to the differing intensity of solar UV radiation, which can kill the bacilli. A number of studies in different countries indicate that the survival of *M. bovis* in environmental matrices is variable (192). *M. bovis* in faeces or faeces-contaminated soil appears to remain viable for up to ~6 months in some studies (193, 194). More recently, Barbier et al. (195) have undertaken *in vitro* experiments in which differing soil types were seeded with *M. bovis* and incubated at 4 and 22˚C. Their findings indicated that *M. bovis* persisted for longer (up to 150 days) at the cooler temperature, whilst results for differing soil types were inconclusive (195). It may also be worth investigating whether *M. bovis* strain variation may have a role to play in adaptation to environmental persistence. Within the Eu1 major lineage that dominates the UK and Ireland (as discussed above), it has been noted that there is considerable heterogeneity in cell wall content as detected by Fourier Transform Infra-Red Spectroscopy (196). Indeed, the major genetic deletion which is a hallmark of the Eu1 lineage removes a gene responsible for trehalose biosynthesis – an important component of the glycolipid rich hydrophobic cell wall (124). Recently it has been observed that hydrophobic cell wall components, which are a feature of the pathogenic bacilli in the *Mycobacterium tuberculosis* complex, aid aerosolisation and pathogenicity (197). Conversely, environmental mycobacteria appear to have more hydrophilic cell wall components (197). Could the Eu1 lineage, or some of its descendants have evolved a phenotype that retained pathogenicity but permitted environmental persistence? Comparison to other lineages in the type of experiments Barbier et al. (195) have performed may be a useful way of addressing this hypothesis.

### Current and Future Climate Effects on *M. Bovis* Persistence in the Environment

Also pertinent to this debate is the climate of the UK and Ireland compared to continental Europe. Britain and Ireland inhabit a zone of the globe whose predominant weather tends to be mild and wet without experiencing extremes in temperature – classified under the Köppen system as a Cfb climate; temperate with no dry season and 10 or more months of the year exhibiting temperatures above 10 ˚C (198). Much of Western Europe, including northern Spain, most of France, Germany, Belgium and Holland are also categorised as belonging to this Köppen climate category (198). However, it is noted that whilst the Köppen system is useful for broad inferences of year on year regional climate (199), it can miss intra-regional variation, particularly in European locales (198). Britain and Ireland are a case in point. Both territories are islands, surrounded on all sides by the Atlantic Ocean and various seas. Indeed, the North Atlantic Oscillation is the primary driver of the maritime climate niche within the broader Cfb category that Britain and Ireland occupy (200). Whilst there is variation across Britain and Ireland in climate - a general west to east cline in temperature, precipitation and sunlight is observed (200) - the general trend (even for south east Britain, which is most like the continent) is that both islands exhibit milder winters and cooler summers than continental neighbours (201). Indeed, records show that these islands receive less sunshine (202) and more precipitation (203) than other Western European countries. Given what we have discussed above about the factors effecting viability of *M. bovis* in the environment, could these contemporary conditions influence *M. bovis* survival in the environment of these islands, or specific regions of them, compared to continental nations? The obvious counterpoint to this is that Britain in particular came close to eradicating bTB through the 1950 and 1960s (18). So, if a contaminated environment was important for regional persistence, shouldn’t that have prevented the scale of decline in prevalence during that period? It depends on the likely scale of importance an environmental reservoir would constitute, and whether that importance has changed with time. Perhaps the changing circumstances in wildlife abundance, farming practices, strain effects etc since the 1970s could have conspired to make a contaminated environment more of a contemporary problem in contrast to the past? Alternatively, if the role of environmental contamination is relatively small regardless of the point in time it occurs in, then it could still be an important factor in regional persistence. For instance, Britain did not completely eradicate bTB after initial success in reducing prevalence throughout the 1950 and 1960 s. Low level infection in cattle remained a problem with eventual recrudescence through the 1970–2000 s leading to the current impasse (18). It is plausible that an environmental reservoir may have played a role, alongside other factors, in preventing that final push to complete eradication.

With changing climate in the future, the UK and Ireland are predicted to see even milder, wetter weather (204, 205), with intra-regional variation in exact outcomes. Predicted general trends are for drier, hotter summers (205, 206), potentially with less cloud cover in southern Britain (205), and milder, wetter winters with increased probability of extreme precipitation events [Sweeney et al, 2001; (205)]. It would be pertinent therefore to begin to address whether contemporary climate effects and predicted future effects are likely to have any impact on the survivability of *M. bovis* in the British and Irish environment. These questions could perhaps be addressed in the future using field data and *in vitro* experimentation. The effect of weather conditions have been correlated with variation in *M. bovis* risk in cattle (116, 207–209), and such weather variation has significant impact on the population dynamics of wild reservoirs also (74, 210) potentially impacting patterns of infection (95), adding to the complexity.

### Emerging Environmental Hosts

Alongside general environmental contamination with *M. bovis*, and potentially contributing to it, is the role that soil based organisms may play in dissemination of bacilli and their persistence. Specifically, protozoa have been implicated as potential reservoirs of *M. bovis*. It has in the past been hypothesised that the benign environmental bacteria that went on to become virulent, intracellular pathogens, may have evolved many of their intracellular persistence apparatus within the “nurseries” of environmental protozoa (211–214). Initially, Mardare et al. (215) had suggested that amoeba predation of bacilli was more likely to result in inactivated bacilli and reduced persistence in environmental samples. However, more recently it has been shown that protozoa containing TB causing bacilli, when fed to mice can result in active tuberculous infection (216).

Earthworms have also recently been observed to ingest *M. bovis* from cattle faeces and disseminate bacilli in castings across the wider landscape (217). From regional sampling and regression of soil content data, predicted earthworm abundance and species diversity across Europe have recently been determined (218). These data demonstrate that earthworm abundance is greatest in Denmark, Holland, Britain and Ireland compared to other Western European countries and that Ireland and Britain display one of the highest diversities of species across the continent (218). Earthworms have also been noted as a major component of the diet of badgers, particularly in Britain (219) and to a lesser extent in Ireland (220). Greater investigation of the potential role these ecosystem engineer species play in the epidemiology of bTB may shed light on environmental persistence and transmission dynamics.

### What About Scotland? Is It the Exception That Proves the Rule?

Scotland poses an apparent challenge to the paradigm we have attempted to develop within this manuscript as an Officially TB Free (OTF) territory within Britain – it is part of the British isles, badgers reside there (there are also other potential wildlife hosts, with relatively large deer populations) especially in the lowlands, there is a significant cattle industry, and similar tests and testing regimes have been employed as in the rest of the British Isles. However, the relative magnitude of these characteristics is worth dwelling upon.

Badger density is significantly lower than in the rest of the British Isles (66, 67, 69, 70, 221). Badger abundance can be estimated using the density of main setts, representing the number of social groups within an area, allowing for reasonable estimation of abundance at large national scales (222). Comparing the mean social group density across countries of the British Isles, Scotland has the lowest mean density of 0.11 social groups km^−2^ (to the nearest thousand, 9,000 social groups (221), whereas in England and Wales the average value is 0.49 km^−2^ [72,000 social groups (66)], the Republic of Ireland has 0.25 km^−2^ [(19,000 social groups (69)]; and Northern Ireland, with the highest estimated density, of 0.58 km^−2^ [8,000 social groups (70)]. While there is debate as to the linearity of the relationship between social group density and abundance [(223); but see (222)] the magnitude of the difference would suggest a significant difference in average badger population density between the countries (69). This is most likely related to the low proportion of the most suitable badger habitat in Scotland (much of Scotland is exposed, and/or upland).

Similarly, Scotland’s cattle density is significantly lower than other countries within the British Isles [see above (6)]. However, the cattle industry is concentrated in the lowlands, meaning that similar intensity of farming may be occurring at local scales, with large herd sizes being reported (6). Scotland has a lower proportion of dairy farms, relative to the rest of the UK and ROI, and dairy herds can represents an increased risk relative to other farming types [(6, 224); but see (225)].

 Furthermore, there are significant trade links between Scotland and the rest of the UK and ROI, including from relatively high TB risk areas (225, 226). This trade represents a risk for Scottish farmers who trade in from high incidence areas (225, 226), but the relative risk has been diminished significantly through the introduction of pre- and post-movement testing of traded animals (226). These restrictions have reduced the amount of international trade and also reduced the risk to infection (re)introduction (226).

In terms of liver fluke, recent spatial models would suggest that much of Scotland (especially the highlands) is high risk and future climate changes may exacerbate this problem (174). The confounding effects of co-infection in low prevalence situations seem not to mitigate against the maintenance of disease freedom.

Scotland is on a quadrennial testing regime, with herds tested at least once every four years (225), therefore, the testing regime is at a lower frequency than much of the rest of the UK and Ireland.

An argument could be made that Scotland reduced historic bTB levels to a low enough level to allow cattle measures alone to achieve freedom. Perhaps then, the historical reduced wildlife and cattle densities were then sufficient to act as a bulwark against recrudescence and the establishment of endemic disease? In fact, GB was very close to eradication in the 1980s before bTB re-emerged significantly in the 30 years since (18). During this period high bTB levels were largely confined to the south and south east of Britain, from where the epidemic has slowly expanded (227). In the intervening period badger population density has increased significantly (66–68), the intensity of cattle farming may also have increased, coinciding with policy changes, and there may be potential interactive effects of concurrent infections, all of which would have been exacerbated by the 2001 foot and mouth crisis (6).

## Conclusions

In this article we have attempted to propose potential reasons why the British and Irish experience in eradicating bovine tuberculosis has been so fraught compared to that of other jurisdictions in recent times. Our suggestions have arisen from a broad comparative approach which contrasts landscape, ecological, animal husbandry and molecular epidemiological characteristics within Britain and Ireland to those primarily observed on the wider European Continent. We note, with caution, that correlation is not causation. However for all proposed factors, we have endeavoured to present a coherent narrative, supported by published evidence, which links each to pertinent aspects of bTB epidemiology. We do not propose that these potential factors are exhaustive, merely that they may be worthy of further investigation, and individually or collectively may constitute novel hypotheses that go some way to explaining the comparative lack of progress in bTB eradication in these islands.

Our hypothesis is that owing to their history, ecology and geography, Britain and Ireland may occupy a “goldilocks zone” for bovine TB. Factors highlighted in this review include the presence of a sufficient wildlife reservoir, a potentially amenable environment for *M. bovis* maintenance, a number of endemic infections that could impact on the diagnosis and transmission of bTB, an evolutionary lineage of the pathogen unique to Western Europe and a large, highly connected, dense network of farms where the movement of infected animals could be facilitated, partially due to the limitations of the statutory test at the individual level.

As regards further investigation, we propose a wider scale comparison of all listed factors across Britain and Ireland, and their association with risk of bTB persistence and other pertinent epidemiological outcomes, contrasted to territories / regions with lower bTB prevalence. The latter may help to ascertain if any of the factors have a significant impact on bTB eradication efforts and also to quantify their relative importance. The latter type of investigation could be achieved in two ways:

1. Aggregating all retrospective information for the listed factors across multiple patches of interest across Britain and Ireland into a single data resource that could contrast intra-regional differences and find potential associations and effects – in effect a meta-analysis.
2. Prospectively, across Britain, Ireland and Western European countries, identify regions with varying burdens of disease and actively measure / catalogue the stated factors for statistical analyses.

A caveat is that both strategies would bring their own inherent problems. Both would require harmonisation of retrospectively and prospectively collected data, to control for differences in bTB eradication scheme administration and data collection methods.

However, efforts to survey broader vistas of the bTB landscape may make these efforts more worthwhile, identifying novel mechanisms amenable to control. There may have been a tendency to restrict one’s horizons when investigating bTB persistence in Britain and Ireland – a parochial approach, that whilst understandable with a complex disease affecting many herds and animals on a national scale – may miss some important epidemiological drivers. Owing to potential intra-national homogeneity in the characteristics of risk factors, their relative importance at a wider scale could be masked – for example: since the lineage of *M. bovis* found in Britain and Ireland lacks diversity, intra-national comparison of potential effects of strain variation would be difficult to detect, since everything seems so genetically similar.

A potential criticism of our focus on some of these factors, is that even if they did have a significant effect on bTB epidemiology, that effect may be very small and therefore, any intervention would potentially not be practical or cost efficient. However, in the absence of firm evidence either way, this criticism could appear to be somewhat pessimistic. The reproductive index (R0) for bovine TB between cattle in Britain has been estimated to be low – 1.1 (228). Between badgers, R0 has also been observed to be low – ranging from 1.03 to 1.19 (223). Between species R0 has recently been estimated to be in the region of 0.05 (60). These results suggest that not much effort may be needed to tip the R0 (of the two-host system) below one and drive the epidemic to extinction. It may well be that targeted intervention on multiple factors of small effect, when combined with the larger effects of the nationally managed eradication schemes, could help achieve this goal. In effect, we are suggesting that addressing some of the potential factors identified here, may result in an aggregation of marginal gains that takes the standard eradication scheme protocol as its base line, and applies an ecosystem management approach to drive down remaining infection.

## Author Contributions

AA and AB came up with the concept for the manuscript and drafted it. RS made additions to the text, edited existing text and gave advice.

## Conflict of Interest Statement

The authors declare that the research was conducted in the absence of any commercial or financial relationships that could be construed as a potential conflict of interest.
