# Corrigendum: Bovine Tuberculosis in Britain and Ireland – A Perfect Storm? the Confluence of Potential Ecological and Epidemiological Impediments to Controlling a Chronic Infectious Disease

**DOI:** 10.3389/fvets.2019.00213

**Published:** 2019-07-02

**Authors:** A. R. Allen, R. A. Skuce, A. W. Byrne

**Affiliations:** Veterinary Science Division, Agri-Food and Biosciences Institute, Belfast, United Kingdom

**Keywords:** *Mycobacterium bovis*, Britain and Ireland, eradication, persistence, epidemiology

In the original article, there was an error. The tipping point of R0 was given as “0,” while it should have stated “1.”

A correction has been made to the **Conclusions** section, paragraph five:

“A potential criticism of our focus on some of these factors, is that even if they did have a significant effect on bTB epidemiology, that effect may be very small and therefore, any intervention would potentially not be practical or cost efficient. However, in the absence of firm evidence either way, this criticism could appear to be somewhat pessimistic. The reproductive index (R0) for bovine TB between cattle in Britain has been estimated to be low – 1.1 (228). Between badgers, R0 has also been observed to be low – ranging from 1.03 to 1.19 (223). Between species R0 has recently been estimated to be in the region of 0.05 (60). These results suggest that not much effort may be needed to tip the R0 (of the two-host system) below one and drive the epidemic to extinction. It may well be that targeted intervention on multiple factors of small effect, when combined with the larger effects of the nationally managed eradication schemes, could help achieve this goal. In effect, we are suggesting that addressing some of the potential factors identified here, may result in an aggregation of marginal gains that takes the standard eradication scheme protocol as its base line, and applies an ecosystem management approach to drive down remaining infection.”

The authors apologize for this error and state that this does not change the scientific conclusions of the article in any way. The original article has been updated.

